# Palmitic Acid Downregulates Thyroglobulin (Tg), Sodium Iodide Symporter (NIS), and Thyroperoxidase (TPO) in Human Primary Thyrocytes: A Potential Mechanism by Which Lipotoxicity Affects Thyroid?

**DOI:** 10.1155/2018/4215848

**Published:** 2018-10-17

**Authors:** Meng Zhao, Xiaohan Zhang, Ling Gao, Yongfeng Song, Chao Xu, Chunxiao Yu, Shanshan Shao, Jiajun Zhao

**Affiliations:** ^1^Department of Endocrinology, Shandong Provincial Hospital Affiliated to Shandong University, Jinan, Shandong 250021, China; ^2^Shandong Provincial Key Laboratory of Endocrinology and Lipid Metabolism, Jinan, Shandong 250021, China; ^3^Institute of Endocrinology and Metabolism, Shandong Academy of Clinical Medicine, Jinan, Shandong 250021, China; ^4^Scientific Center, Shandong Provincial Hospital Affiliated to Shandong University, Jinan, Shandong 250021, China

## Abstract

Our previous studies suggested that the thyroid might be a target organ affected by lipotoxicity, which might be partially related to the increasing prevalence of subclinical hypothyroidism. However, the underlying molecular mechanism is not yet clearly established. This study aimed to assess the effect of palmitic acid stimulation on thyrocyte function. Upon palmitic acid stimulation, intracellular contents of lipids, as well as the expression and activity of three key molecules in thyroid hormone synthesis (i.e., thyroglobulin, sodium iodide symporter, and thyroperoxidase), were determined in human primary thyrocytes. The contents of BODIPY® FL C16 (the fluorescently labeled palmitic acid analogue) entering into the thyrocytes were gradually increased with time extending. Accordingly, the intracellular accumulation of both triglyceride and free fatty acids increased in dose- and time-dependent manners. The effect of palmitic acid stimulation on thyroid hormone synthesis was then determined. Both the mRNA and protein levels of thyroglobulin, sodium iodide symporter, and thyroperoxidase were decreased following palmitic acid stimulation. Consistently, upon palmitic acid stimulation, the secreted thyroglobulin levels in supernatants, ^131^I uptake, and extracellular thyroperoxidase activity were all decreased in a dose-dependent manner. Our results demonstrated that upon palmitic acid stimulation, the expressions of the key molecules (thyroglobulin, sodium iodide symporter, and thyroperoxidase) were reduced and their activities were suppressed, which might lead to impaired thyroid hormone synthesis.

## 1. Introduction

Hypothyroidism, which is defined as failure of thyroid gland to produce sufficient thyroid hormone to meet the metabolic demands of the body, does affect a considerable proportion of the population [1–4]. A survey carried out in 10 cities in China indicated that the prevalence of hypothyroidism had dramatically increased from 4.33% in 1999 to 17.73% in 2011 [5]. It is universally accepted that primary hypothyroidism can result from congenital abnormalities, autoimmune destruction (Hashimoto disease), iodine deficiency, iatrogenic injury, and infiltrative diseases, among which autoimmune thyroid disease is the most common etiology of primary hypothyroidism [6]. However, risk factors contributing to the increasing prevalence of hypothyroidism have not been fully confirmed.

Lipotoxicity has attracted great attention worldwide for its serious and extensive impact on human health. In recent years, lipotoxicity has been well documented in the pathogenesis of type 2 diabetes mellitus, metabolic syndrome, and nonalcoholic fatty liver disease [7, 8]. Furthermore, in the early 1990s, lipotoxicity induced by dietary fat overload was reported to interfere with the endocrine system [9]. Our previous studies suggested that the thyroid might be another target organ affected by lipotoxicity, which might be partially related to the increasing prevalence of subclinical hypothyroidism [10, 11]. However, the underlying molecular mechanism is not yet clearly established.

Here, we explored the effect of palmitic acid (PA) stimulation on thyrocyte. This study provides a more comprehensive understanding of the pathophysiological effects of lipotoxicity on thyroid function.

## 2. Materials and Methods

### 2.1. Human Primary Thyrocyte Culture

Human thyroid samples were obtained from patients undergoing partial thyroidectomy for treatment of benign follicular nodules at Shandong Provincial Hospital affiliated to Shandong University from December 2016 to May 2017. Both men and women were adopted and each individual underwent serum thyroid function and antibody testing and Doppler ultrasonography. Subjects with thyroid tumor, thyroid dysfunction, and autoimmune thyroid disease were all excluded. The Ethics Committee of Shandong Provincial Hospital approved the experimental protocol, and all participants provided written informed consent before collecting samples. According to the standardized procedure [12], all of the obtained specimens were dissected from the region most distant from the affected tissue and were digested in 2 mg/ml collagenase I (Sigma-Aldrich) and 0.125% trypsin for 1 hour. After filtering and washing, thyrocytes were seeded on culture dishes (Corning). The medium consisted of DMEM/F12 medium (HyClone) supplemented with 10% newborn calf serum (GIBCO), bovine TSH (2 mU/ml; Sigma-Aldrich), and penicillin-streptomycin (100 U/ml). The cells were cultured in a humidified incubator containing 95% air and 5% CO_2_ at 37°C until cell attachment. The culture medium was replaced with fresh medium every 24 hours. As appropriate for the experiments to be performed, the cells were washed twice with PBS and then starved in serum-free medium overnight before they were treated with palmitic acid (Sigma-Aldrich).

### 2.2. Intracellular Localization of BODIPY® FL C_16_

Human primary thyrocytes were washed with PBS and incubated with 1 *μ*M BODIPY® FL C_16_ (Life Technologies), the fluorescently labeled palmitic acid analogue in 0.1% DMSO, pH 7.2 at 37°C for 5 minutes, 15 minutes, and 30 minutes, respectively. Then, the thyrocytes were briefly rinsed with PBS and fixed with 4% paraformaldehyde for 15 min at 37°C. Nuclei were stained with 4, 6-diamidino-2-phenylindole (DAPI; Vector Laboratories) and the resultant immunofluorescence was viewed under a fluorescent microscope (Axio Imager 2, Carl Zeiss, Jena, Germany).

### 2.3. Determination of Intracellular Triglyceride and Free Fatty Acid

Human primary thyrocytes were incubated in DMEM/F12 medium containing 10% newborn calf serum until 80% confluency was achieved. The cells were subsequently changed to serum-free medium and treated without or with different concentrations of palmitic acid (0.2 or 0.4 mM) for the indicated time intervals (12 or 48 hours). The intracellular triglyceride and free fatty acid was extracted and measured using a Triglyceride Quantization Kit (Applygen Technologies) and a Free Fatty Acid Quantization Kit (Biovision) according to the manufacturer's instructions. All data were normalized with the protein concentration in a parallel well.

### 2.4. Quantitative Real-Time PCR

Quantitative real-time PCR was performed according to a previously described method [13]. Total RNA from cells was isolated using Trizol reagent (Takara, Tokyo, Japan), following the manufacturer's instructions. RT reaction (20 *μ*l) was carried out using 1 ug of total RNA, oligo-dT primer, random 6-mers, and reverse transcriptase (RT) (Takara). Real-time PCR was performed in LC480 (Roche, Mannheim, Germany) according to instructions. SYBR green (Takara) was used to detect the amplification of cDNA in a total volume of 20 *μ*l with the absolute quantitative, ΔCt method. Each reaction consisted of 10 *μ*l of SYBR green, 1 *μ*l of cDNA sample, 2 *μ*l of each primer pair (5 *μ*M), and 7 *μ*l of distilled water. Thermal cycling conditions were 5 min at 95°C followed by 40 cycles at 95°C for 10 sec, 60°C for 10 sec, and 72°C for 10 sec. The PCR primers were shown in Table 1. *β*-Actin was employed as an endogenous control to normalize the data. The specificity of the PCR amplification was verified by melting curve analysis of the final products (cooling the sample to 60°C and heating slowly to 95°C with measurement of fluorescence) and 10 *μ*l loaded on a 3% agarose gel to determine the size and specificity of the PCR product. All data were relative to control.

### 2.5. Western Blot Analysis

Equal amounts of protein from different samples were subjected to 10% SDS-PAGE, followed by electrotransfer from the gel to polyvinylidene difluoride membranes (Millipore). The membranes were incubated overnight at 4°C with anti-NIS (1 : 250, Biorbyt), anti-TPO (1 : 200, Santa Cruz), anti-Tg (1 : 10000, Abcam), and anti-BiP (1 : 1000, Proteintech). GAPDH protein was evaluated as a loading control (1: 3500, Proteintech). Immune complexes were detected using the FluorChem Q Gel Imaging and Analysis System (Protein Simple, California, USA), and band densitometry was performed through the use of associated densitometry software (Protein Simple, California, USA).

### 2.6. Immunofluorescence

Human primary thyrocytes were treated without or with 0.2 mM palmitic acid for 72 hours. The cells were washed with PBS and fixed with 4% paraformaldehyde. Then, the cells were washed and blocked with 10% goat serum and incubated with anti-NIS (1 : 100, Biorbyt), anti-TPO (1 : 100, Santa Cruz), and anti-Tg (1 : 50, Abcam) at 4°C overnight, respectively. After further washing, the thyrocytes were incubated with secondary TRITC- or FITC-conjugated goat anti-mouse IgG. Nuclei were stained with 4, 6-diamidino-2-phenylindole (DAPI; Vector Laboratories) and the resultant immunofluorescence was viewed under a fluorescent microscope (Axio Imager 2, Carl Zeiss, Jena, Germany). All images were acquired using the same exposure time. As a negative control, the identical procedure was performed in the absence of the primary antibody and replaced by PBS. The semiquantitative analysis of fluorescence intensity was conducted using Image J software.

### 2.7. Detection of Secretory Thyroglobulin (Tg)

Human primary thyrocytes were seeded in 6-well plates and treated without or with palmitic acid (0.2 or 0.4 mM) for 72 hours. The secretory Tg levels in supernatants were determined using chemiluminescent methods (Cobas E601; Roche, Basel, Switzerland) and normalized with corresponding protein amount.

### 2.8. Radioactive Iodide Uptake Assay

Human primary thyrocytes were seeded in 6-well plates and treated without or with palmitic acid (0.2 or 0.4 mM) for 72 hours. Radioactive iodide uptake assay was performed according to Zhang et al. previously described [14]. Briefly, cells were washed twice with Hanks' balanced salt solution and incubated with 2.0 *μ*Ci Na^125^I (Chengdu Gaotong Isotype Co. Ltd.) in 5 *μ*M nonradioactive NaI (Sigma-Aldrich) for 30 minutes at 37°C with 5% CO_2_. The cells incubated in the presence of 80 *μ*M NIS inhibitor, perchlorate (Sigma-Aldrich), were determined simultaneously as blank controls. Cells were then washed twice with cold Hanks' balanced salt solution and lysed with 95% ethanol for 20 min. The cell lysate was collected and radioactivity was counted by a *γ*-counter (MN-6110, Zonkia, Anhui, China). Radioactive iodide uptake values were normalized with corresponding cell counts. Experiments were performed in triplicate.

### 2.9. Enzymatic Activity of Extracellular TPO

Considering that TPO is expressed at the surface of the cell, we determined its extracellular enzymatic activity as previously described [15]. Human primary thyrocytes were seeded in 6-well plates and treated without or with palmitic acid (0.2 or 0.4 mM) for 72 hours. The cells were washed twice with 2 ml of PBS, then 0.5 ml of reaction mixture was added (100 *μ*M KI (Sigma-Aldrich), 200 U/ml superoxide dismutase (SOD, Sigma-Aldrich), and 50 *μ*M Amplex Red (Life Technologies) in sodium phosphate buffer). The reaction was initiated by adding 25 *μ*l of 1 mM H_2_O_2_ (Sigma-Aldrich). 20 *μ*l of aliquots were removed at 1-minute intervals during 8 minutes and immediately mixed with 80 *μ*l of inhibition mixture containing 500 U/ml catalase (Sigma-Aldrich) and 100 U/ml SOD in PBS. The fluorescence was measured in a microtiter reader (Mithras^2^ LB 943, Berthold, Bad Wildbad, Germany) using excitation at 530 nm and emission at 590 nm. Enzymatic activity values were normalized with corresponding protein amount.

### 2.10. Cell Viability Assay

Cell viability was assessed by Cell Counting Kit-8 (Dojindo, Japan) assay [16, 17]. Cells were seeded at 10000 cells/well into 96-well plates with 90 *μ*l culture medium. The 10 *μ*l of CCK-8 solution was added to the cells at specific time points and cells were incubated for 2 hr at 37°C. The absorbance was measured at 450 nm. The reaction product was quantified according to the manufacturer's instructions.

### 2.11. Statistical Analyses

Statistical tests were performed using SPSS version 18.0 for Windows (Chicago, IL, USA). Differences among control group and palmitic acid treatment groups were compared using one-way analysis of variance (ANOVA) (LSD or Dunnett's *t*-test) for multiple comparisons. The differences of extracellular TPO activity among control group and palmitic acid treatment groups were compared using repeated measurement data ANOVA. All of the calculated *P* values were two sided, and *P* values less than 0.05 were considered to be statistically significant.

## 3. Results

### 3.1. Excess Lipid Accumulation Induced by PA Stimulation in Human Primary Thyrocytes

**Figure 1:**
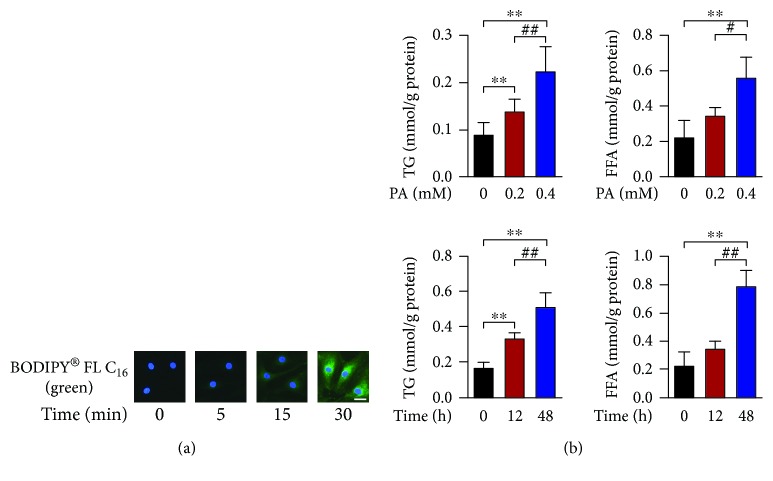
Palmitic acid increases the intracellular triglyceride and free fatty acid contents in human thyrocytes. (a) Intracellular distribution of BODIPY® FL C_16_, the fluorescently labeled palmitic acid analogue (1 *μ*M) in cells with time extending. Scale bars, 20 *μ*m. (b) Upper: dose-dependent intracellular accumulations of triglyceride and free fatty acid in cells exposed to palmitic acid for 24 hours (*n* = 4). Lower: time-dependent intracellular accumulations of triglyceride and free fatty acid in cells exposed to palmitic acid at the concentration of 0.2 mM (*n* = 3). All data are expressed as the mean ± standard deviation. ^∗∗^ *P* < 0.01 versus human primary thyrocytes without palmitic acid stimulation. ^#^ *P* < 0.05 and ^##^ *P* < 0.01 versus human primary thyrocytes with palmitic acid stimulation at 0.2 mM or for 12 hours. The error bars represent the standard deviations.

PA, the most abundant saturated fatty acid found in diets and bloodstream, is well known to induce lipotoxic effect in various nonadipose cells and often employed to study the potential molecular mechanisms of cellular damage generated by lipotoxicity [18]. We determined whether PA was able to enter the human primary thyrocytes, which might be a prerequisite for lipotoxicity. As shown in Figure 1(a), the contents of BODIPY® FL C_16_ (the fluorescently labeled PA analogue) entering into the thyrocytes were gradually increased with time extending. Accordingly, the intracellular accumulation of both triglyceride and free fatty acids increased in dose- and time-dependent manners (Figure 1(b)).

### 3.2. Expression Suppression of Key Molecules Involved in Thyroid Hormone Synthesis Induced by PA Stimulation in Human Primary Thyrocytes

**Figure 2:**
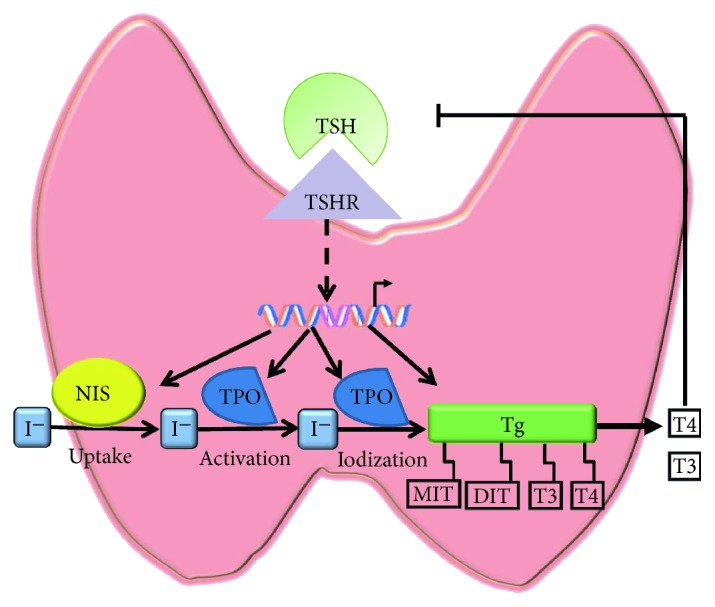
The process of thyroid hormone synthesis.

**Figure 3:**
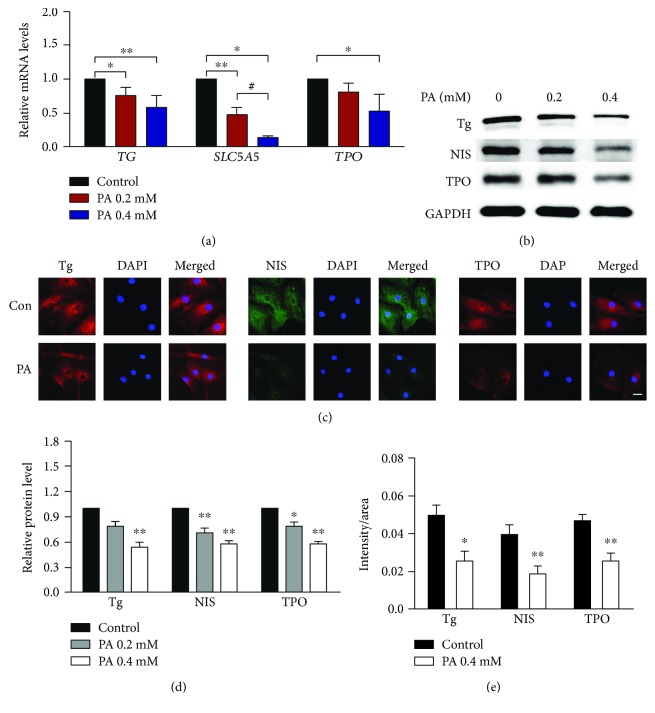
Palmitic acid downregulates the expression of thyroglobulin (Tg), sodium iodide symporter (NIS), and thyroperoxidase (TPO) in human primary thyrocytes. (a) The mRNA levels of Tg, NIS (SLC5A5), and TPO in cells stimulated with palmitic acid for 24 hours (*n* = 3 to 5). (b) Dose-dependent effects of palmitic acid on the protein levels of Tg, NIS, and TPO for 72 hours detected by Western blot. (c) The protein levels of Tg, NIS, and TPO detected by immunofluorescence in cells stimulated with palmitic acid at 0.2 mM for 72 hours. Scale bars, 20 *μ*m. (d) The relative protein levels from Western blot assays for Tg, NIS, and TPO was quantified by densitometry and normalized with GAPDH. (e) The relative fluorescence intensity from immunofluorescence assays for Tg, NIS, and TPO. In all panels, representative data from 3 to 7 independent experiments are shown. All data are expressed as the mean ± standard deviation. ^∗^ *P* < 0.05 and ^∗∗^ *P* < 0.01 versus human primary thyrocytes without palmitic acid stimulation; ^#^ *P* < 0.05 versus human primary thyrocytes stimulated with palmitic acid at 0.2 mM. The error bars represent the standard deviations. PA: palmitic acid; Tg: thyroglobulin; SLC5A5: gene name of sodium iodide symporter; TPO: thyroperoxidase.

Tg, NIS, and TPO are all key molecules in thyroid hormone synthesis (Figure 2) [19]. As shown in Figure 3(a), the mRNA levels of these three molecules decreased with the stimulation of PA. Moreover, their protein levels were also decreased significantly in human primary thyrocytes following PA stimulation (Figures 3(b) and 3(c) and Supplementary Figure 1), and the differences reached statistical significance (Figures 3(d) and 3(e)).

### 3.3. Activity Suppression of Key Molecules Involved in Thyroid Hormone Synthesis Induced by PA Stimulation in Human Primary Thyrocytes

**Figure 4:**
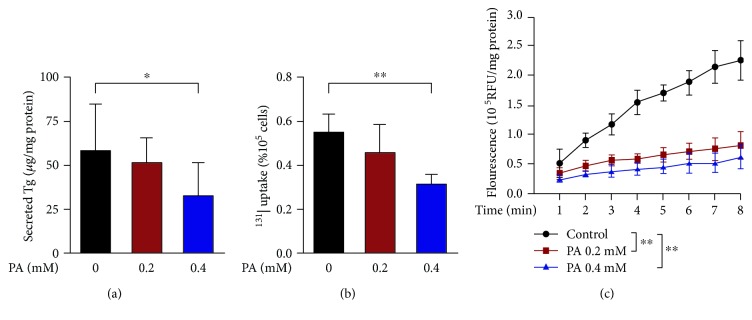
Palmitic acid downregulates the activity of thyroglobulin (Tg), sodium iodide symporter (NIS), and thyroperoxidase (TPO) in human primary thyrocytes. (a) Secreted Tg levels in supernatants of cells stimulated with palmitic acid for 72 hours (*n* = 7). The Tg levels were normalized with corresponding protein amount. (b) The effects of palmitic acid stimulation on ^131^I uptake for 72 hours (*n* = 7). ^131^I uptake values were normalized with corresponding cell counts. (c) Extracellular TPO activity of the cells stimulated with palmitic acid for 72 hours (*n* = 6). Enzymatic activity values were normalized with corresponding protein amount. In all panels, representative data from 3 to 7 independent experiments are shown. All data are expressed as the mean ± standard deviation. ^∗^ *P* < 0.05 and ^∗∗^ *P* < 0.01 versus human primary thyrocytes without palmitic acid stimulation. The error bars represent the standard deviations. PA: palmitic acid; RFU: relative fluorescence unit.

As shown in Figure 2, Tg secretion, ^131^I uptake, and TPO catalysis are primary procedures in thyroid hormone synthesis. Consistently, in addition to the decreased expression, the activity of the three key molecules was also declined upon PA stimulation. As shown in Figure 4, the secreted Tg levels in supernatants and ^131^I uptake were both decreased by approximately 30% in human primary thyrocytes stimulated with 0.4 mM PA. Also, extracellular TPO activity was decreased in a dose-dependent manner.

### 3.4. Cell Viability Was Not Influenced by PA Stimulation in Human Primary Thyrocytes

To exclude the possibility that the reduction in expression and activity of key molecules was due to cell death, we performed cell viability assay. As shown in Supplementary Figure 2, cell viability was not influenced in human primary thyrocytes stimulated with 0.2 mM PA (*P* = 0.361) or 0.4 mM PA (*P* = 0.657) at any time point. Therefore, the reduced expression and activity of key molecules was not attributed to cytotoxicity.

### 3.5. Endoplasmic Reticulum Stress Was Induced by PA Treatment in Human Primary Thyrocytes

To explore the involved mechanism by which PA affects the three key molecules, we further observed the expression of binding immunoglobulin protein (BiP), a master regulator of unfolded protein response. As shown in Supplementary Figure 3, the mRNA and protein levels were both obviously increased upon PA stimulation in human primary thyrocytes. These results indicated that endoplasmic reticulum stress was induced by PA treatment in human primary thyrocytes.

## 4. Discussion

Our results demonstrated that PA could induce excess intracellular lipid accumulation in human thyrocytes. Moreover, upon PA stimulation, the expressions of the key molecules were reduced and their activities were suppressed, which might lead to impaired thyroid hormone synthesis. Our study preliminarily suggests the involved mechanism by which lipotoxicity affects thyroid.

The main function of thyroid gland is to concentrate iodide and to make it available for biosynthesis of triiodothyronine (T3) and its precursor thyroxine (T4) [20]. In this process, a number of thyroid hormone synthesis-related molecules were involved, such as Tg, NIS, and TPO (Figure 2). NIS, locating in the basolateral membrane of follicular epithelial cells, transports iodate from bloodstream into the epithelial cells. Iodide enrichment mediated by NIS is the first step of thyroid hormone synthesis [21, 22]. TPO is located at the top of follicular epithelial cells. It catalyzes several crucial reactions, including activation of iodine, iodination of tyrosine residues, and coupling of iodinated tyrosine [19]. Furthermore, the main function of iodinated tyrosine on Tg is to provide precursor substances for thyroid hormone synthesis, and it is also the storage form of thyroid hormone [19, 23]. Since the above three molecules are involved in thyroid hormone synthesis, their suppressed expression and/or activity will decrease serum thyroid hormone concentration and finally lead to thyroid dysfunction.

Our previous studies demonstrated the effect of lipotoxicity on thyroid. In population, we found the positive association between hypertriglyceridemia and risk for subclinical hypothyroidism [10]. In rats, the high-fat lard diet decreased serum total T4 and free T4 levels in parallel with elevated serum thyroid-stimulating hormone levels [11]. However, the potential mechanism has not been fully elucidated. The present work suggested that lipotoxicity may induce hypothyroidism by suppressing the expression and/or activity of thyroid hormone synthesis, which is in agreement with some other studies. For instance, Xia et al. showed that NIS, TPO, and Tg mRNA levels were suppressed in obesity-prone mice fed a high-fat diet [24]. Interestingly, in Lee MH's research, although serum and intrathyroidal thyroxine levels were lower than those fed with normal chow diet, the expression of three key molecules was increased in diet-induced obese mice. This seemingly paradoxical elevation was speculated to result from the stimulatory actions of upregulated thyroid-stimulating hormone [25]. The above inconsistent observations suggest that further investigation is needed to clarify the complicated changes in thyroid with the stimulation of lipotoxicity.

Our study may provide novel clues for in-depth investigations to determine the possibly involved mechanism by which lipotoxicity affects thyroid. In *Wen et al.*'s study, endoplasmic reticulum stress was demonstrated to inhibit expression of Tg, NIS, and TPO in FRTL-5 thyrocytes [26]. Since PA was also found to cause endoplasmic reticulum stress, we further investigated the change of BiP expression. Its upregulation indicated that endoplasmic reticulum stress was triggered in human primary thyrocytes stimulated by PA treatment. Similarly, in our animal experiments, endoplasmic reticulum stress was observed to be activated in male Sprague-Dawley rats fed with high-fat diet (data not shown). Thus, endoplasmic reticulum stress might mediate the effects of PA treatment on the suppression of three key molecules. In addition, sterol regulatory element-binding proteins (SREBPs), which were initially identified as master transcriptional regulators of lipid biosynthesis and uptake, were also reported to be novel transcriptional regulators of Tg, NIS, and TPO in thyroid epithelial cells [27–29]. Other mechanism, such as mitochondrial oxidative stress or inflammation was also possible to be involved in the pathological process.

In summary, our study illustrated the suppressed expressions and activity of the key molecules (i.e., Tg, NIS, and TPO) in thyroid hormone synthesis upon PA stimulation. It is a preliminary exploration focusing on the mechanism by which lipotoxicity affects thyroid, and it provides novel clues for further investigations.

## Figures and Tables

**Table 1:** Primers for quantitative real-time PCR in the study.

	NM	Product (bp)	Forward (5′ to 3′)	Reverse (5′ to 3′)
Human NIS	000453.2	180	GTCCTTCAGGGCTCCTTCACC	CTGCTCGCTGGGTGGGTACA
Human TPO	000547.5	241	CTGTCTGTCACGCTGGTTATGG	TCACTCCGCTTGTTGGCTCA
Human Tg	003235.4	203	TTCTTTGAATGTGAACGACGGTG	AAGGGATAGGTGTGGACTTCAATGT
Human BiP	005347.4	150	GCCTGTATTTCTAGACCTGCC	TTCATCTTGCCAGCCAGTTG
Human *β*-actin	001101	104	ACAGAGCCTCGCCTTTGCCG	ACATGCCGGAGCCGTTGTCG

NIS: sodium iodide symporter; TPO: thyroperoxidase; Tg: thyroglobulin; BiP: binding immunoglobulin protein.

## Data Availability

The data used to support the findings of this study are included within the supplementary information file(s).

## Supplementary Materials

Supplementary Materials Figure S1: palmitic acid time-dependently downregulates the protein levels of thyroglobulin (Tg), sodium iodide symporter (NIS), and thyroperoxidase (TPO). Figure S2: cell viability was not influenced by palmitic acid stimulation in human primary thyrocytes. Figure S3: palmitic acid upregulates the expression of binding immunoglobulin protein (BiP) in human primary thyrocytes. For all the readers to access the data supporting the conclusions of our study, we provided primary data of all the figures in the manuscript here. The means and standard deviations, shown in the figures, were both evaluated based on these data.
