# Supplementary material for: Palmitic Acid Downregulates Thyroglobulin (Tg), Sodium Iodide Symporter (NIS), and Thyroperoxidase (TPO) in Human Primary Thyrocytes: A Potential Mechanism by Which Lipotoxicity Affects Thyroid?
Source: Int J Endocrinol. 2018 Oct 17;2018:4215848. doi: 10.1155/2018/4215848 (PMC6207890; doi:10.1155/2018/4215848)
Supplement: Supplementary Materials — Figure S1: palmitic acid time-dependently downregulates the protein levels of thyroglobulin (Tg), sodium iodide symporter (NIS), and thyroperoxidase (TPO). Figure S2: cell viability was not influenced by palmitic acid stimulation in human primary thyrocytes. Figure S3: palmitic acid upregulates the expression of binding immunoglobulin protein (BiP) in human primary thyrocytes. For all the readers to access the data supporting the conclusions of our study, we provided primary data of all the figures in the manuscript here. The means and standard deviations, shown in the figures, were both evaluated based on these data. [file 4215848.f1.pdf]

Supplementary Figure 1

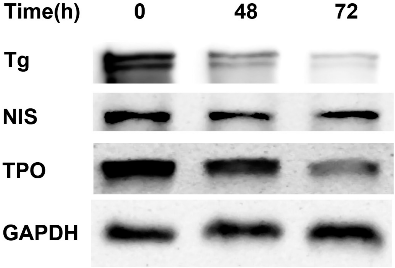

Supplementary Figure 2

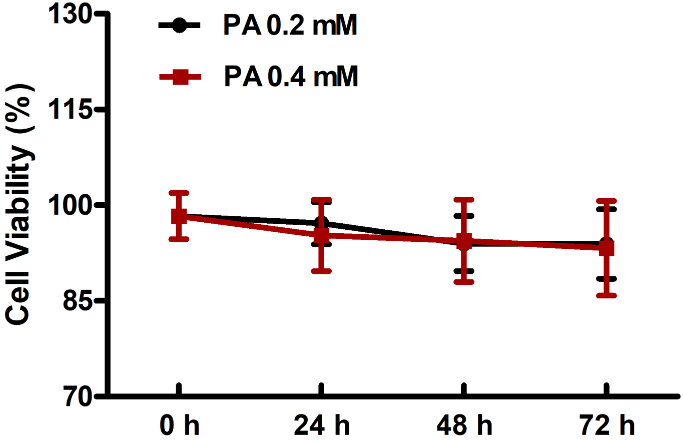

Supplementary Figure 3

A

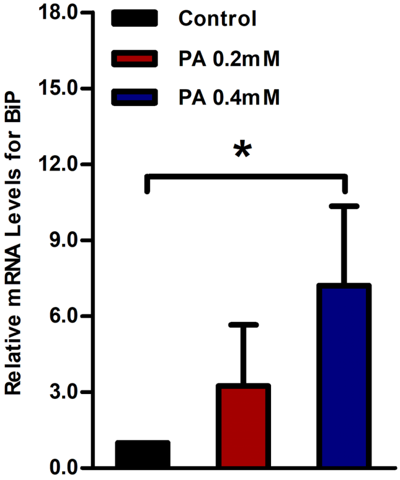

B

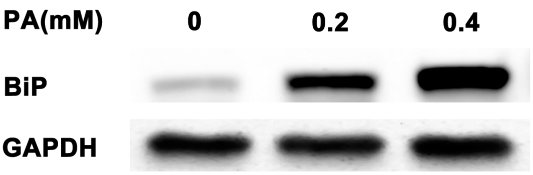

Supplementary Figure Legends

Supplementary Fig. 1. Palmitic acid time-dependently down-regulates the protein levels of thyroglobulin (Tg), sodium iodide symporter (NIS) and thyroperoxidase (TPO). The data are observed in the cells with the palmitic acid stimulation at the concentration of 0.2 mM for 72 hours. PA, palmitic acid.

Supplementary Fig. 2. Cell viability was not influenced by palmitic acid stimulation in human primary thyrocytes. The data are observed in the cells with the palmitic acid stimulation at the concentration of 0.2 mM and 0.4mM for 24hours, 48hours and 72 hours, respectively (n=5). All data are expressed as the mean ± standard deviation. PA, palmitic acid.

Supplementary Fig. 3. Palmitic acid up-regulates the expression of binding immunoglobulin protein (BiP) in human primary thyrocytes. (A) The mRNA levels of BiP in cells stimulated with palmitic acid for 24 hours (n = 3). The data are expressed as the mean ± standard deviation. *P < 0.05 versus human primary thyrocytes without palmitic acid stimulation. The error bars represent the standard deviations. (B) Dose-dependent effects of palmitic acid on the protein levels of BiP for 72 hours detected by western blot. PA, palmitic acid; BiP, binding immunoglobulin protein.

Figure 1 B

Dose-dependent Effect			
	PA Treatment (mM)		
TG	0	0.2	0.4
	0.1235955	0.1794872	0.2816902
	0.06854532	0.1380177	0.1524778
	0.06319115	0.1030338	0.159464
	0.1113714	0.1490733	0.2571467
	0.06852839	0.1379758	0.2549621
	0.08700101	0.1187774	0.2245361
	PA Treatment (mM)		
FFA	0	0.2	0.4
	0.20496	0.398914	0.673184
	0.322745	0.316328	0.550077
	0.118373	0.2988	0.445025

Time-dependent Effect			
	PA Treatment (h)		
TG	0	12	48
	0.127621	0.36541	0.424142
	0.202201	0.301469	0.562999
	0.133451	0.309	0.54296
	PA Treatment (h)		
FFA	0	12	48
	0.20496	0.398914	0.761676
	0.322745	0.316328	0.908996
	0.118373	0.2988	0.671789

Figure 2 A

	PA Treatment(mM)		
	0	0.2	0.4
Tg	1	0.659754	0.447513
	1	0.895025	0.779165
	1	0.712025	0.543367
SLC5A5	1	0.441352	0.146604
	1	0.381565	0.105843
	1	0.632878	0.156041
	1	0.438303	0.136201
TPO	1	0.846745	0.320857
	1	0.574349	0.183011
	1	0.846745	0.702223
	1	0.907519	0.702223
	1	0.870551	0.716978

Figure 2 D

	PA Treatment(mM)		
	0	0.2	0.4
Tg	0.9999992	0.8034703	0.4798879
	0.9999992	0.8245	0.5263
	0.9999992	0.7243	0.6042
NIS	0.9999897	0.7428215	0.519510
	0.9999897	0.7296	0.6023
	0.9999897	0.6588	0.5838
TPO	0.999998	0.7675418	0.5722955
	0.999998	0.7884	0.6041
	0.999998	0.8365	0.5431

Figure 2 E

	PA Treatment(mM)	
	0	0.4
Tg	0.050000	0.027000
	0.055000	0.0300
	0.045000	0.0200
NIS	0.039000	0.020000
	0.035000	0.0220
	0.045000	0.0140
TPO	0.047000	0.025000
	0.044000	0.0220
	0.050000	0.0300

Figure 3 A

	PA Treatment(mM)		
	0	0.2	0.4
Tg	30.67	21.74	1.78
	33.45	41.77	12.94
	37.48	42.60	18.47
	37.97	43.66	30.71
	45.53	46.57	34.09
	47.46	50.76	40.79
	48.96	53.29	44.38
	50.56	62.17	53.49
	65.72	63.74	56.33
	88.82	67.54	
	103.43	70.56	
	105.00		

Figure 3 B

	PA Treatment(mM)		
	0	0.2	0.4
I^{131} Uptake	0.60	0.36	0.33
	0.48	0.32	0.29
	0.45	0.34	0.33
	0.66	0.57	0.36
	0.53	0.62	0.27
	0.57	0.52	

Figure 3 C

	PA Treatment(mM)			
		0	0.2	0.4
Fluorescence	1	0.2446053	0.4621396	0.3252412
		0.4124483	0.3838636	0.2393845
		0.5538421	0.2746364	0.1987071
		0.4488179	0.2504202	
		0.4530096		
		0.9530232		
	2	0.8775105	0.5762688	0.3975705
		0.9107258	0.5178859	0.322144
		0.9148666	0.3945227	0.2932696
		0.8612037	0.3770108	
		0.7184623		
		1.084631		

	3	0.9982252	0.5932095	0.495427
		1.179743	0.6577726	0.3569379
		1.267890	0.4959545	0.294899
		1.233315	0.4839136	
		0.9280476		
		1.358708		
	4	1.358501	0.5978318	0.5405145
		1.443937	0.6934712	0.3951226
		1.816478	0.5406591	0.341587
		1.701155	0.5354742	
		1.303313		
		1.592143		
	5	1.575012	0.5707648	0.5853162
		1.699150	0.8185017	0.4431664
		1.752443	0.6542727	0.3424017
		1.901531	0.5781913	
		1.523622		
		1.734794		
	6	1.684353	0.5927567	0.7037156
		1.670946	0.8891414	0.4664841
		2.061579	0.7282727	0.3768686
		2.170768	0.6342937	
		1.769348		
		1.913468		
	7	1.812307	0.5793424	0.699732
		1.795269	1.038997	0.4498957
		2.365619	0.7625682	0.4152852
		2.421091	0.642437	
		2.147572		
		2.295348		
	8	1.823050	0.5874911	0.8572169
		1.915939	1.132312	0.5362285
		2.456747	0.8112273	0.4876018
		2.700349	0.7115646	
		2.351290		
		2.276068		

Supplementary Figure 2

		PA Treatment (mM)	
		0.2	0.4
Cell Viability	0h	93.7323	93.7323
		97.15102	97.15102
		103.6912	103.6912
		99.23198	99.23198
		97.596940	97.59694
	24h	99.38062	98.1915
		96.7051	89.27311
		101.4616	90.31358
		93.13774	95.81326
		95.2187	102.7993
	48h	93.58366	101.1643
		101.1643	93.58366
		93.58366	101.0157
		89.57039	87.93535
		92.09727	88.52991
	72h	89.42175	86.44895
		90.61086	102.948
		91.05679	94.02958
		95.81326	85.40847
		102.6507	97.4483

Supplementary Figure 3A

	PA Treatment (mM)		
	0	0.2	0.4
BiP	1	1.310393404	4.055837919
	1	2.496661098	7.210003701
	1	5.938094283	10.33882265
